# Dual Expression of the *Salmonella* Effector SrfJ in Mammalian Cells and Plants

**DOI:** 10.3389/fmicb.2017.02410

**Published:** 2017-12-06

**Authors:** Julia Aguilera-Herce, Azhar A. Zarkani, Adam Schikora, Francisco Ramos-Morales

**Affiliations:** ^1^Departamento de Genética, Facultad de Biología, Universidad de Sevilla, Sevilla, Spain; ^2^Julius Kühn-Institut – Bundesforschungsinstitut für Kulturpflanzen, Federal Research Centre for Cultivated Plants, Institute for Epidemiology and Pathogen Diagnostics, Brunswick, Germany

**Keywords:** *Salmonella*, SrfJ, type III secretion, *myo*-inositol, macrophages, plants, bioluminescence

## Abstract

SrfJ is an effector of the *Salmonella* pathogenicity island 2-encoded type III secretion system. *Salmonella enterica* serovar Typhimurium expresses *srfJ* under two disparate sets of conditions: media with low Mg^2+^ and low pH, imitating intravacuolar conditions, and media with *myo*-inositol (MI), a carbohydrate that can be used by *Salmonella* as sole carbon source. We investigated the molecular basis for this dual regulation. Here, we provide evidence for the existence of two distinct promoters that control the expression of *srfJ*. A proximal promoter, P*srfJ*, responds to intravacuolar signals and is positively regulated by SsrB and PhoP and negatively regulated by RcsB. A second distant promoter, P*iolE*, is negatively regulated by the MI island repressor IolR. We also explored the *in vivo* activity of these promoters in different hosts. Interestingly, our results indicate that the proximal promoter is specifically active inside mammalian cells whereas the distant one is expressed upon *Salmonella* colonization of plants. Importantly, we also found that inappropriate expression of *srfJ* leads to reduced proliferation inside macrophages whereas lack of *srfJ* expression increases survival and decreases activation of defense responses in plants. These observations suggest that SrfJ is a relevant factor in the interplay between *Salmonella* and hosts of different kingdoms.

## Introduction

*Salmonella enterica* serovar Typhimurium (*S.* Typhimurium) is a facultative intracellular bacterial pathogen that can survive and proliferate in diverse hosts (Wiedemann et al., 2014) as well as non-host environments (Winfield and Groisman, 2003). This non-typhoidal *Salmonella* serovar can infect a broad range of animal species. In humans, it causes gastrointestinal infections with occasional secondary bacteremia (Chen et al., 2013). In mice, in contrast, it produces systemic infections that are similar to typhoid fever induced by *S. enterica* serovar Typhi in humans (Garai et al., 2012). It also causes acute enteritis and exudative diarrhea in calves, which are considered as a relevant model for non-typhoidal salmonellosis in humans (Costa et al., 2012). In addition, chickens and pigs can be asymptomatic carriers of these bacteria (Foley et al., 2007). As a consequence, these animals are important sources of *S.* Typhimurium in the food chain. *S.* Typhimurium and other serovars can also enter the agricultural production chain. This can happen on different levels, e.g., via animal feces used for soil amendments or as post-harvest contamination. *Salmonella* is able to adhere to plant surface, colonize plant organs, and suppress the plant immune system (Schikora et al., 2012; Neumann et al., 2014). Therefore, plants are considered as alternative hosts for these pathogens, and fresh fruits and vegetables are recognized as an important source of food-borne disease (Wiedemann et al., 2014; Holden et al., 2015).

Many virulence factors are involved during the interactions of *Salmonella* with its host. Prominent among them are two type III secretion systems (T3SS), heteromultimeric nanomachines specialized in the delivery of effector proteins into host cells (Deng et al., 2017). More than 30 effectors are translocated by *Salmonella* into the host cell through both T3SSs (Ramos-Morales, 2012).

Effectors secreted by the *Salmonella* Pathogenicity Island 1 (SPI1)-encoded system (T3SS1) (Galán and Curtiss, 1989) promote invasion of host cells by a trigger mechanism that involves remodeling the actin cytoskeleton to form membrane ruffles that internalize the bacteria in a vacuole known as *Salmonella*-Containing Vacuole (SCV). This system is also involved in modulation of epithelial tight junctions (Boyle et al., 2006; Liao et al., 2008; Zhang et al., 2015; Lin et al., 2016), induction of polymorphonuclear leukocytes transepithelial migration (Zhang et al., 2006; Wall et al., 2007), control of the initial stages of the SCV biogenesis (Bakowski et al., 2008; Steele-Mortimer, 2008), inhibition of host cell exocytosis (Perrett and Zhou, 2013), and induction of rapid pyroptosis in macrophages (Fink and Cookson, 2007). The SPI2-encoded system (T3SS2) is expressed several hours after internalization and is important for SCV biogenesis, intracellular survival and proliferation, generation of *Salmonella*-induced tubules, and systemic infection (Hensel, 2000; Knodler and Steele-Mortimer, 2003; Liss and Hensel, 2015).

SrfJ is a poorly characterized T3SS2 effector (Cordero-Alba et al., 2012). Interestingly, virulence of a *S.* Typhimurium strain with mutation in *srfJ* was mildly attenuated in mice (Ruiz-Albert et al., 2002). The SrfJ protein shares 30% amino acid sequence identity with human glucosylceramidase over 447 residues (Kim et al., 2009). Initially, it was proposed as a putative effector because the gene *srfJ* is positively regulated by SsrB (Worley et al., 2000), the main positive regulator of T3SS2 (Fass and Groisman, 2009). Surprisingly, we found that, in addition to the regulation by SsrB, *srfJ* is also negatively regulated by IolR (Cordero-Alba et al., 2012), the repressor of the *myo*-inositol (MI) utilization genes in *S.* Typhimurium (Kröger and Fuchs, 2009). *Salmonella* and other bacteria can use MI as the sole carbon source, and this substrate is ubiquitous in soil and plants.

Here, we analyzed the molecular basis and studied the ecological relevance of the dual regulation of *srfJ*. We found that two distinct promoters control the expression of *srfJ*. The proximal (P*srfJ*) responds to intravacuolar signals in animal cells, whereas the second and distant P*iolE* is active during plant colonization.

## Materials and Methods

### Bacterial Strains, Bacteriophages, and Strain Construction

*Escherichia coli* and *S.* Typhimurium strains used in this study are described in **Table 1**. *Salmonella* strains derived from the mouse-virulent strain ATCC 14028. Transductional crosses using phage P22HT 105/1 *int201* (Schmieger, 1972) were used for strain construction (Maloy, 1990). To obtain phage-free isolates, transductants were purified by streaking on green plates. Green plates were prepared as described previously (Chan et al., 1972), except that methyl blue (Sigma) substituted aniline blue. Phage sensitivity was tested by cross-streaking with the clear-plaque mutant P22 H5 (Chan et al., 1972).

**Table 1:** Bacterial strains and plasmids used in this study.

Strain/plasmid	Relevant characteristics	Source/reference
***E. coli*** **strains**		
DH5α	*supE44 ΔlacU169 (Δ80 lacZΔ*M15*) hsdR17 recA1 endA1 gyrA96 thi-1 relA1*	Hanahan, 1983
***S. enterica*** **serovar Typhimurium strains^a^**		
14028	Wild type	ATCC
55130	14028 *pho-24* (PhoP constitutive)	E.A. Groisman
SV4608	14028 *trg*::Mu*d*J Km^r^	Segura et al., 2004
SV4699	14028 *phoP7953*::Tn*10*	Laboratory stock
SV4758	14028 *rcsC55*	García-Calderón et al., 2005
SV5049	14028 Δ*rcsB*::Cm^r^	Laboratory stock
SV5373	14028 Δ*hilA*	Laboratory stock
SV5452	14028 Δ*ssrB*::Cm^r^	García-Calderón et al., 2007
SV5559	14028 Δ*srfJ::lacZ* Km^r^	Laboratory stock
SV5599	14028 *srfJ*::3xFLAG Km^r^	Cordero-Alba et al., 2012
SV6891	14028 Δ*iolR*::Cm^r^	Cordero-Alba et al., 2012
SV9416	14028 ΔP*iolE*::Cm^r^	This study
**Plasmids**		
pGEM-T Easy	Vector for cloning PCR products	Promega
pIZ2182	pSB377-P*iolG1*	This study
pIZ2183	pSB377-P*iolI1*	This study
pIZ2184	pSB377-P*srfJ*	This study
pIZ2185	pSB377-P*iolE*	This study
pIZ2306	pSB377-P*iolE-*P*srfJ*	This study
pKD3	*bla* FRT *cat* FRT PS1 PS2 oriR6K	Datsenko and Wanner, 2000
pKD46	*bla* P_BAD_ *gam bet exo* pSC101 oriTS	Datsenko and Wanner, 2000
pSB377	Parent for *luxCDABE* transcriptional fusions, Ap^r^	Winson et al., 1998


*^a^Derivatives of these strains were used as indicated in the text.*

### Bacterial Culture

The standard culture medium for *S. enterica* and *E. coli* was Luria-Bertani (LB) broth. Solid LB contained 1.5% agar (final concentration). Antibiotics were used at the following concentrations: kanamycin (Km), 50 μg/ml; chloramphenicol (Cm), 20 μg/ml; ampicillin (Ap), 100 μg/ml; and tetracycline (Tc), 20 μg/ml. For some experiments, 55.5 mM MI was added for 4 h. For SPI1-inducing conditions, *Salmonella* strains were grown overnight at 37°C in LB 0.3 M NaCl medium in static conditions. For SPI2-inducing conditions, cells from cultures in LB were washed and diluted 1:125 with minimal medium at pH 5.8 (LPM) containing 80 mM 2-(*N*-morpholino)-ethanesulfonic acid (pH 5.8), 5 mM KCl, 7.5 mM (NH_4_)_2_SO_4_, 0.5 mM K_2_SO_4_, 0.1% Casamino acids, 38 mM glycerol, 337.5 μM K_2_HPO_4_–KH_2_PO_4_ (pH 7.4), and 8 μM MgCl_2_, and then incubated overnight at 37°C with shaking. Two different media with plants extracts were made: Lettuce Medium (LM) contained 25% lettuce plant extract sterilized with 0.22 μm filter and 20% M9-Minimal Salts (Sigma) (Fornefeld et al., 2017); Tomato Medium (TM) contained 25% tomato plant extract sterilized with 0.22 μm filter and 20% M9-Minimal Salts. *Salmonella* cells from saturated cultures in LB were washed with MgCl_2_ 10 mM and diluted in each plant media at OD_600_ 0.1, and then incubated at 37°C with shaking. All the experiments involving *S.* Typhimurium were carried out using the standard biosecurity procedures that include containment level 2 practices, and safety equipment and facilities.

### DNA Amplification with the PCR

Amplification reactions were carried out in a T100 Thermal Cycler (BioRad). For plasmid constructs, the final volume of reactions was 50 μl, and the final concentration of MgCl_2_ was 1.5 mM. Reagents were used at the following concentrations: deoxynucleoside triphosphates (dNTPs), 300 μM; primers, 0.3 μM; and Taq polymerase (KAPA HiFi DNA Polymerase; Kapa Biosystems), 1 U per reaction. The PCR program included the following steps: (i) initial denaturation for 5 min at 95°C; (ii) 25 cycles of denaturation (98°C, 20 s), annealing (57°C, 15 s), and extension (72°C, 30 s); and (iii) final incubation at 72°C for 5 min to complete the extension. For colony PCR, the final volume of reactions was 20 μl. Reagents were used at the following concentrations: 1× MyTaq Red Reaction Buffer; primers, 0.3 μM; and Taq polymerase (MyTaq Red DNA Polymerase; Bioline), 1 U per reaction. The program included the following steps: (i) initial denaturation for 3 min at 95°C; (ii) 25 cycles of denaturation (95°C, 15 s), annealing (57°C, 15 s), and extension (72°C, 30 s–2 min); and (iii) final incubation at 72°C for 5 min to complete the extension. Primers are listed in **Table 2**. PCR constructs were sequenced with an automated DNA sequencer (Stab Vida, Oeiras, Portugal) to confirm that the sequence was correct.

**Table 2:** Oligonucleotides used in this study.

Oligonucleotide/use	Sequence (5′–3′)
**Construction of pIZ2182**	
PiolG1ecofw	GTTCGAATTCCATGCCGCTACTGAGTAAAC
PiolG1ecorev	ATGCGAATTCTTAAAGTCATTTTCTGTTTCC
**Construction of pIZ2183**	
PiolI1ecofw	CTGAGAATTCTGACATGATTGGTAATTTCAAATC
PiolIecorev	ATGCGAATTCTCAGATCGACTCCTGCCGCC
**Construction of pIZ2184**	
PsrfJecofw	ATGCGAATTCTCACTGCGATGTTACCGGCG
PsrfJecorev	TGCAGAATTCAGGGAAGTTCCGGATAAAAGAAG
**Construction of pIZ2185**	
PiolEecofw	GTCAGAATTCTCAATATCGCAAGGACTATC
PiolEecorev	CTGAGAATTCTGGCTCCCACTTAATGAAAC
**Construction of pIZ2306**	
PiolEecofw	GTCAGAATTCTCAATATCGCAAGGACTATC
PsrfJecorev	TGCAGAATTCAGGGAAGTTCCGGATAAAAGAAG
**Deletion of P*iolE***	
PiolEH1P1fw	TTCAGAATTACTTCAAAAATAAAGTAGGGAAAAC
	GCCCGGGTGTAGGCTGGAGCTGCTTC
PiolEH2P2rev	ATCCCCAACTTAATGCTCTTTTTTACATTGTAC
	ATATTGCCATATGAATATCCTCCTTAG
**qRT-PCR**	
CHI3fw	TGCAGGAACATTCACTGGAG
CHI3rev	TAACGTTGTGGCATGATGGT
CHI9fw	GAAATTGCTGCTTTCCTTGC
CHI9rev	CTCCAATGGCTCTTCCACAT
GLUAfw	GGTCTCAACCGCGACATATT
GLUArev	CACAAGGGCATCGAAAAGAT
GLUBfw	TCTTGCCCCATTTCAAGTTC
GLUBrev	TGCACGTGTATCCCTCAAAA
PR-1afw	TCTTGTGAGGCCCAAAATTC
PR-1arev	ATAGTCTGGCCTCTCGGACA
actinfw	AGGCACACACAGGTGTTATGGT
actinrev	AGCAACTCGAAGCTCATTGT
**RT-PCR**	
Efw	GGGCATCAATATTCTGGCTG
G1fw	ACCCTCAAAACCTGATTTCTAC
G1rev	TTAAGTGATCGGAGCCGATC
Jrev2	GACGATGCGAAAAAGAGACC
Jfw	CAGACTCATCTCTTCCGATC
Jrev	CATGCTGTTGAATACCACGC
Irev	AAACGTTCCGCCAACACAAC
Jfw2	GATGTCCAGGAAAGGCGTTG
**5′-RACE**	
5RaceNested	GACACTGACATGGACTGAAGGA
Erev	GAAACAACGGCGACATATGC
Erev2	ATCATTGCGCCAACCGATAG
Jrev	CATGCTGTTGAATACCACGC
Jrev2	GACGATGCGAAAAAGAGACC
Gene Racer RNA Oligo	UGGAGCACGAGGACACUGACAU
	GGACUGAAGGAGUAGAAA


### Plasmids

Plasmids used in this study are listed in **Table 1**. Plasmid pSB377 (Winson et al., 1998) was used to make transcriptional fusions of putative promoter regions with the *luxCDABE* operon from *Photorhabdus luminescens*. This operon encodes a bacterial luciferase whose product, the light, can be measured without disturbing the cell or adding any substrate. To make these constructions, DNA from strain 14028 was used as a template for PCR amplification with the primers listed in **Table 2**. The amplified fragments were digested with EcoRI and ligated with EcoRI digested and dephosphorylated pSB377. The ligation mixture was transformed into *E. coli* DH5α and transformants were selected in LB agar supplemented with Ap. Transformants with plasmids containing the correct transcriptional *lux* fusions were isolated and verified by PCR and sequencing (Stab Vida, Oeiras, Portugal).

### Generation of a P*iolE* Mutant

Disruption and replacement of P*iolE* with a Cm resistance gene were performed as described previously (Datsenko and Wanner, 2000). Briefly, the Cm resistance gene from plasmid pKD3 was PCR amplified with primers PiolEH1P1fw and PiolEH2P2rev (**Table 2**). The PCR product was used to transform the wild-type strain carrying the Red recombinase expression plasmid pKD46.

### Mammalian Cell Culture

RAW264.7 cells (murine macrophages; ECACC No. 91062702) were cultured in Dulbecco’s modified Eagle medium (DMEM) supplemented with 10% fetal calf serum and 2 mM L-glutamine. The 60 μg/ml penicillin and 100 μg/ml streptomycin were included in the culture media (except for bacterial infection experiments). Cells were maintained in a 5% CO_2_ humidified atmosphere at 37°C.

### Luminescence Measurements and Infection of Cultured Cells

*Salmonella* strains were grown in triplicate in the media described above and samples of 150 μl of each culture were used to measure luminescence and OD_600_. Luminescence was read in white, clear bottom 96-well plates (Corning) using a Synergy HT microplate reader (BioTek) or a Sunrise reader (Tecan). To measure luminescence of intracellular bacteria, RAW264.7 cells were plated into white, clear bottom, 96-well plates at 3 × 10^4^ cells per well, and were infected 24 h later with non-invasive bacteria. For that purpose, bacteria were grown in LB medium for 24 h at 37°C with shaking and were added at a multiplicity of infection (MOI) of 500. Bacteria were centrifuged onto the cell monolayer at 200 g for 5 min and then incubated at 37°C with 5% CO_2_. The cell culture was washed twice with phosphate-buffered saline (PBS) 30 min post-infection (p.i.), overlaid with DMEM containing 100 μg/ml gentamicin, and incubated for 1 h and 30 min. The culture was then washed twice with PBS, covered with DMEM with gentamicin (16 μg/ml), and incubated for 24 h. Luminescence was measured 2, 4, 8, and 24 h p.i. and the numbers of CFU per well were calculated after incubation with 1% Triton X-100 in PBS for 10 min at 37°C to release bacteria, plating appropriate dilutions in LB with Ap, and counting colonies after 24 h of incubation at 37°C. For proliferation assays, infections were carried out using a mix of two strains, as indicated in the Section “Result.” Competitive index for proliferation was calculated as previously described (Segura et al., 2004) after plating appropriate dilutions and enumerating colonies of both strains. Bacteria were recovered 1.25 h p.i. (input) and 24 h p.i. (output).

### RNA Extraction and Reverse Transcription

Bacterial strains were grown overnight in LPM or LB. Thereafter, 4 ml of each strain were pelleted and resuspended in 100 μl of water containing 3 mg/ml lysozyme. RNA from these lysates was isolated with 1 ml of TRIzol reagent (Invitrogen) by using the protocol supplied by the manufacturer. An additional step of phenolization or the kit Direct-zol^TM^ RNA MiniPrep Plus (Zymo Research) was carried out to obtain pure samples. RNA (∼1 μg) was reverse transcribed into cDNA with the Quantitect Reverse Transcriptase (Qiagen) before carrying out PCR with appropriate primers.

### 5′-RACE

Fifteen micrograms of RNA was used to determine the cDNA 5′-end (Bensing et al., 1996; Saito et al., 2009). RNAs were prepared either with or without RNA 5′-Pyrophosphohydrolase (RppH) (New England BioLabs) to distinguish primary transcript 5′-ends from internal 5′-processing sites. DNA primers Jrev and Erev were used for cDNA synthesis with SuperScript III Reverse Transcriptase (Invitrogen) after fusing the GeneRacer RNA Oligo to the isolated RNA. Additional primers for subsequent PCR amplification of cDNAs were GeneRacer 5′-nested primer, homologous to the adaptor GeneRacer RNA oligo, Erev2 and Jrev2. PCR products that were detected both with and without tobacco acid pyrophosphatase treatment were purified by using a PCR clean-up system kit (Promega) and cloned by using the pGEM-T Easy kit (Promega), and three clones of each candidate were sequenced.

### Western Blotting and Antibodies

Protein lysates were prepared in SDS–PAGE sample buffer. Proteins were separated by SDS–PAGE in 12% polyacrylamide gels and electrophoretically transferred to nitrocellulose filters for Western blot analysis using anti-Flag M2 monoclonal antibodies (1:5000; Sigma) or anti-GroEL polyclonal antibodies (1:30,000; Sigma). Goat anti-mouse IgG IRDye 800CW and goat anti-rabbit IgG IRDye 680RD (LICOR) were used as secondary antibodies. Bands were detected using and Odyssey Fc infrared imaging system (LICOR).

### Plant Cultivation

Tomato (*Solanum lycopersicum*) seeds were surface sterilized in 2% natrium hypochlorite solution (10 ml) for 10 min. The seeds were then washed vigorously six times with sterile distilled water. Seeds were germinated during 1 week in Petri dishes with sterile 0.5× Murashige and Skoog (MS) medium (Sigma). Seedlings were grown in sterile conditions in 0.25× MS medium (Sigma) in a cabinet with a light intensity of 150 μmol × m^2^/s (16 h photoperiod) for a further 2 weeks at 22°C.

### Bacterial Colonization of Tomato Plants

*Salmonella enterica* serovar Typhimurium strain 14028 carrying derivatives of plasmid pSB377 (empty, P*iolE*, P*srfJ*, P*iolE*–P*srfJ*) was used to spray 3-week-old tomato plants grown in sterile conditions. Bacteria were grown 1 day before the infection on LM or TM plates. Three tomato plants were spray-inoculated with bacteria suspended in 10 mM MgCl_2_ at OD_600_ 0.1. Tomato plants were imaged 2 days p.i. with an X-ray film exposed for 48 h.

### Bacterial Survival in Plants

To prepare the bacterial inoculum, bacteria were grown on solid LM and then suspended in 10 mM MgCl_2_ and diluted to an OD_600_ = 0.01. Leaves of tomato and lettuce (*Lactuca sativa*) were syringe infiltrated with bacterial solutions, the inoculated leaf areas were sampled 3 h (day 0), 7 days, and 14 days after the inoculation. Serial dilutions were plated on XLD agar to determine the CFU numbers. The experiments were repeated three times with six plants per experiment.

### Analysis of Defense Gene Expression Using Quantitative Real-Time PCR (qRT-PCR)

Total RNA from plant leaves was extracted with TRIZOL reagent (Ambion) and treated with DNase I (Quanta BioSciences) following the suppliers’ protocols. Poly A-tailed RNA (1 μg) was converted to cDNA using the qScript cDNA Synthesis Kit (Quanta BioSciences) and oligo-dT primers. qRT-PCR reactions were performed in triplicates with the Maxtra SYBR Green Master Mix (Fermentas) and run on a BioRad iCycler according to the manufacturer’s instructions. The primers used for the qRT-PCR are presented in **Table 2**. Relative gene expression was normalized to the expression of actin transcript. Expression levels were compared to the control (10 mM MgCl_2_). Data were processed with the iQ software (BioRad).

### Statistical Analysis

Student’s *t*-test was used to analyze every competitive index against the null hypothesis that the mean is not significantly different from 1. This test was also used to compare mean survival of mutants and wild-type *Salmonella* strains in plants, as well as expression levels of defense response genes after colonization with different *Salmonella* strains. *P*-values of 0.05 or less were considered significant.

## Results

### Identification of Promoter Regions Driving the Expression of *srfJ*

**FIGURE 1:**
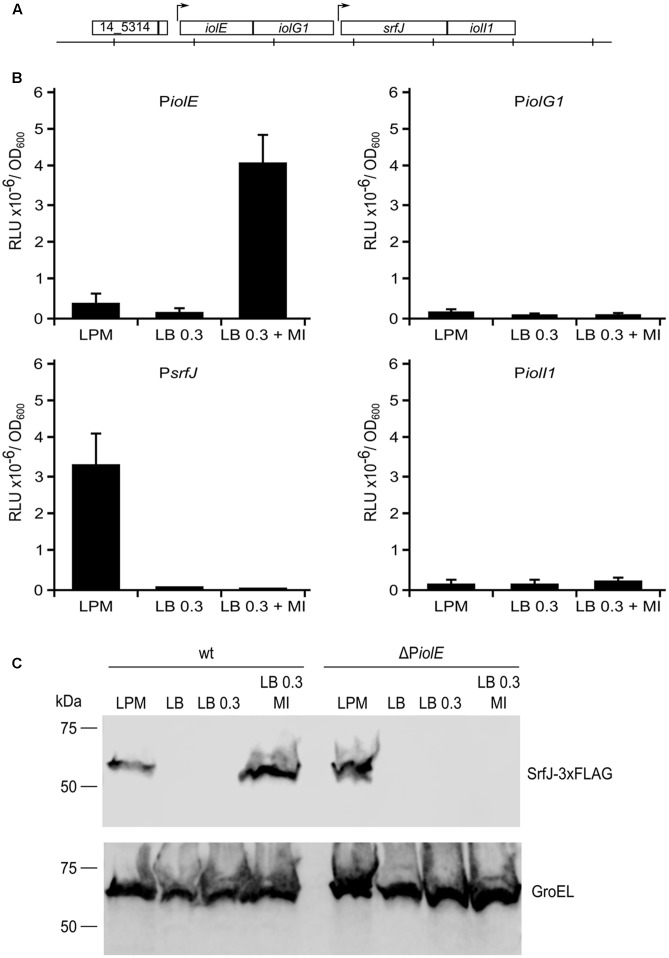
Activity of the putative promoter regions in response to LPM and myo-inositol (MI). **(A)** Representation of the coding regions of *srfJ* and surrounding genes in *S.* Typhimurium strain 14028. The distance between vertical lines represents 1 kb. **(B)** Fragments of DNA upstream the coding regions of genes *iolE*, *iolG1*, *srfJ*, and *iolI1* were cloned into plasmid pSB377 to generate *luxCDABE* transcriptional fusions. These plasmids were introduced into *S.* Typhimurium strain 14028 and luminescence was measured in cultures grown until stationary phase in LPM, LB 0.3 M NaCl, and LB 0.3 M NaCl with MI. RLU, relative light units. **(C)** Extracts of a derivative of *S.* Typhimurium 14028 expressing 3xFLAG-tagged SrfJ were resolved by 12% SDS–PAGE. Immunoblotting was performed with monoclonal anti-FLAG antibodies. Anti-GroEL antibodies were used as loading control. Media tested were LPM, LB, LB with 0.3 M NaCl (LB 0.3), and LB 0.3 M NaCl with MI (LB 0.3 MI). Molecular mass markers are indicated on the left.

Previous data showed expression of *srfJ* under two disparate conditions: culture medium imitating the intravacuolar environment (LPM) and culture medium supplemented with MI (Cordero-Alba et al., 2012). To understand this dual expression at the molecular level, we explored the genomic region around the *srfJ* gene. As shown in **Figure 1A**, this gene resides inside the MI utilization island (Kröger and Fuchs, 2009), with *iolE* and *iolG1* upstream and *iolI1* downstream of *srfJ*. Promoter activities for regions upstream of these genes (putatively called P*iolE*, P*iolG1*, P*srfJ*, and P*iolI1*) were tested using plasmid pSB377 (Winson et al., 1998) that carries a promoterless version of the *luxCDABE* operon of *P. luminescens* that encodes the luciferase LuxAB subunits and a fatty acid reductase complex involved in synthesis of the fatty aldehyde substrate for the luminescence reaction (Meighen, 1991). This reporter system allows continuous monitoring of light production without disrupting the bacteria or the infected host. Plasmids were introduced in wild-type *S.* Typhimurium strain 14028 and the luminescence was measured after growth in three different culture conditions: LPM at pH 5.8 with high aeration for SPI2-inducing conditions, LB with 0.3 M NaCl without aeration for SPI1-inducing conditions, and the later medium supplemented with MI to induce expression of the *iol* genes. Only DNA fragments upstream of *iolE* and *srfJ* coding regions showed promoter activity (**Figure 1B**). Interestingly, P*iolE* was specifically active in the presence of MI whereas P*srfJ* was only active upon SPI2-inducing conditions. These results suggest that expression of *srfJ* is driven by two promoters: a distal promoter, P*iolE*, and a proximal promoter, P*srfJ*, depending on the environmental conditions. Additional support for these conclusions was obtained studying the production of a chromosomically tagged version of the protein SrfJ by immunoblot. As shown in **Figure 1C**, SrfJ-3xFLAG was detected in extracts from bacteria grown in minimal LPM medium and in rich LB medium supplemented with MI. In a ΔP*iolE* background, however, the protein was detected only in LPM, confirming that the distal promoter, PiolE, is specifically necessary for MI-dependent induction of *srfJ*.

### Differential Regulation of P*iolE* and P*srfJ*

**FIGURE 2:**
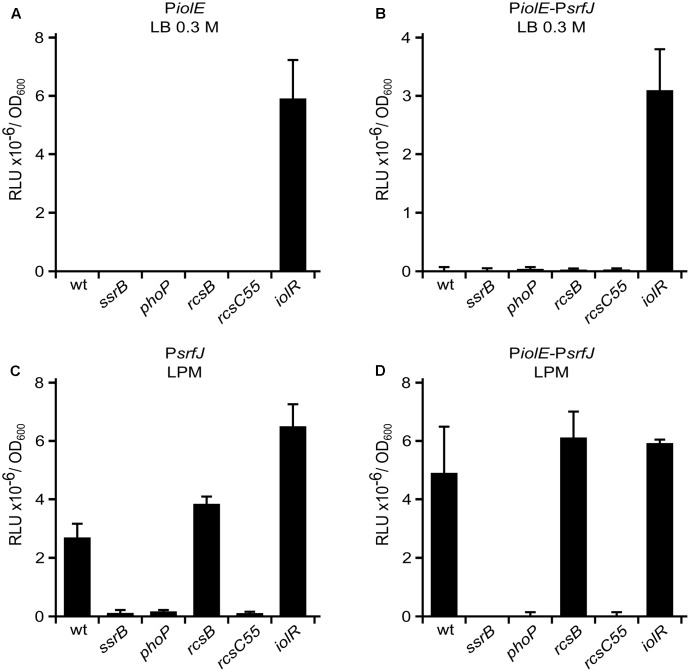
Regulation of P*iolE* and P*srfJ* promoters. Fragments of DNA containing the promoters of *iolE* (P*iolE*) **(A)** or *srfJ* (P*srfJ*) **(C)** or the region containing from the promoter of *iolE* to the promoter of *srfJ*, including genes *iolE* and *iolG1* (P*iolE*–P*srfJ*) **(B**,**D)** were cloned into plasmid pSB377 to generate *luxCDABE* transcriptional fusions. These plasmids were introduced into *S. enterica* serovar Typhimurium strain 14028 or derivatives with null mutations in *iolR*, *ssrB*, *phoP*, *rcsB*, or a point mutation in *rcsC* (*rcsC55*) that confers constitutive activation to the Rcs system. Luminescence was measured in cultures grown until stationary phase in LB 0.3 M NaCl **(A**,**B)** and LPM **(C**,**D)**. RLU, relative light units.

In order to study the regulation of both promoters, the corresponding plasmids were transferred into different genetic backgrounds. We tested the effect of null mutations in genes encoding relevant regulators: IolR, SsrB, PhoP, and RcsB. IolR is the negative regulator of the MI utilization island (Kröger and Fuchs, 2009). SsrB is encoded in SPI2 and is the main positive regulator of the island (Cirillo et al., 1998). PhoP positively regulates SPI2 through SsrB (Bijlsma and Groisman, 2005). RcsB is the response regulator of the Rcs phosphorelay system (Stout and Gottesman, 1990; Chen et al., 2001). In *Salmonella*, it positively or negatively regulates genes in SPI1 and SPI2 depending on the level of activation (Wang et al., 2007; Wang and Harshey, 2009). We also used the allele *rcsC55* that causes constitutive activation of the Rcs system (García-Calderón et al., 2005). Analysis of the expression patterns in the different genetic backgrounds (**Figure 2**) indicates that IolR negatively regulates P*iolE*, whereas P*srfJ* is positively regulated by PhoP and SsrB. In addition, P*srfJ* is also negatively regulated by the Rcs global regulatory system, since the activating mutation *rcsC55* abrogates expression of the *lux* reporter from this promoter. Interestingly, a transcriptional *lux* fusion with 2357 bp upstream of *srfJ* containing both promoters and the intervening genes (P*iolE*–P*srfJ*) is regulated by IolR, PhoP, SsrB, and Rcs, recapitulating the regulation patterns observed with the isolated promoters (**Figures 2B,D**).

### Characterization of Transcriptional Units Containing *srfJ*

**FIGURE 3:**
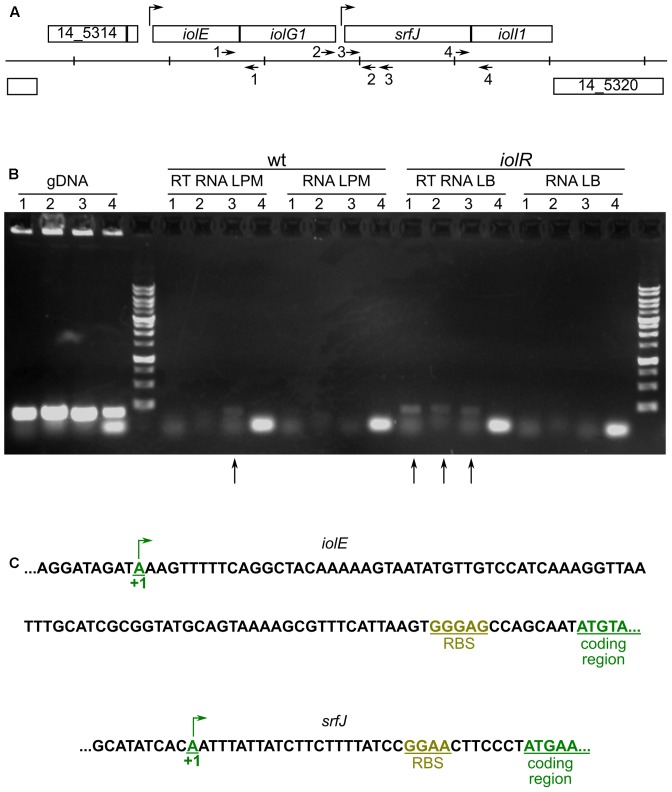
Transcriptional organization of the *srfJ* region. **(A)** Organization of the chromosomal region containing the *srfJ* gene in *S.* Typhimurium strain 14028. Vertical lines are separated by 1 kb. The arrows indicate the positions and orientations of the primers that were used for RT-PCR. **(B)** Agarose gel of the products obtained with the following primers: 1, Efw and G1rev; 2, G1fw and Jrev2; 3, Jfw and Jrev; and 4, Jfw2 and Jrev. RT-PCR was carried out on RNA isolated from cultures on LPM of the wild-type strain (wt, RT RNA LPM) and cultures in LB of the *iolR* mutant strain (*iolR*, RT RNA LB). PCR were also carried out on genomic DNA (gDNA) as positive control and non-retrotranscribed RNA as negative control (RNA LPM and RNA LB). Vertical arrows indicated lanes with amplified products after retrotranscription. The molecular weight marker is the 1 kb DNA ladder (NIPPON Genetics). **(C)** 5′-RACE was carried out on RNA isolated from cultures in LB of the *iolR* mutant to map the transcriptional start site of *iolE* and from cultures in LPM of the wild-type strain to map the transcriptional start site of *srfJ*. The sequences surrounding the transcriptional start sites (+1) and the start of the coding regions are shown. RBS, ribosomal binding site.

Results presented above suggest that two different promoters can initiate the expression of *srfJ*. This would result in RNAs of different lengths. To test this hypothesis, RT-PCR was performed using primers designed to amplify different fragments in the *srfJ* region (**Figure 3A**). RNA was obtained from two sources: (i) wild-type *S.* Typhimurium incubated in LPM, where P*srfJ* is expected to be active, and (ii) *iolR* mutant strain incubated in LB, where the absence of IolR repressor should lead to constitutive expression from P*iolE*. Positive and negative controls were carried out using genomic DNA and non-retrotranscribed RNA, respectively. As seen in **Figure 3B**, RT-PCR carried out on RNA from wild-type bacteria incubated in LPM yielded only an internal fragment of *srfJ*. In contrast, fragments partially expanding *iolE*–*iolG1* and *iolG1*–*srfJ* were obtained when RT-PCR was carried out using RNA from the *iolR* mutant, indicating that these genes are transcriptionally linked when P*iolE* is derepressed. 5′-RACE was used for the determination of both transcriptional start sites. They were located 99 and 33 bp upstream of the coding regions of *iolE* and *srfJ*, respectively (**Figure 3C**). These results confirm that *srfJ* belongs to two different transcriptional units: a short transcriptional unit with P*srfJ* as promoter and an operon including genes *iolE*, *iolG1*, and *srfJ* with P*iolE* as promoter.

### Expression of *srfJ* Inside Macrophages

**FIGURE 4:**
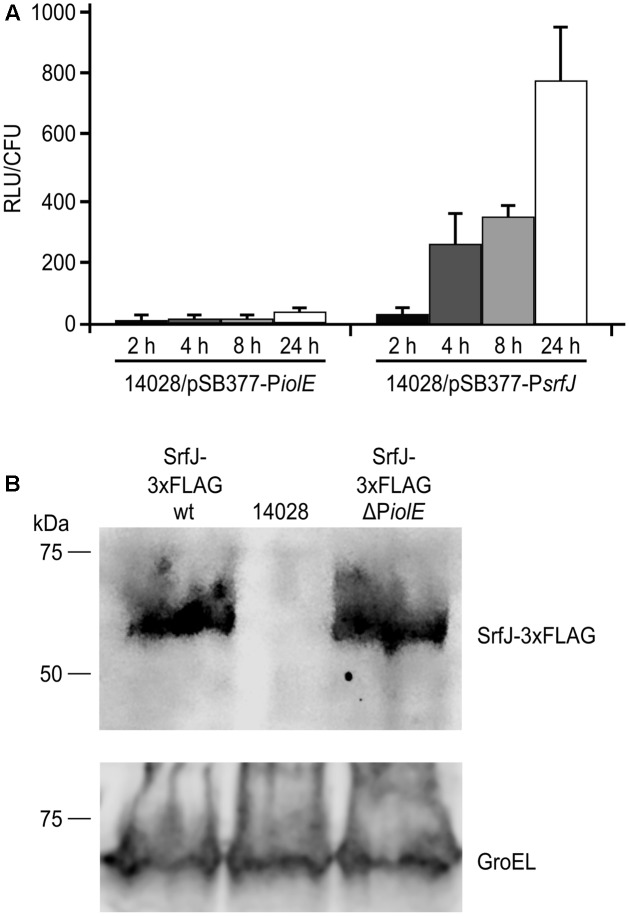
Activity of P*iolE* and P*srfJ* during macrophage infection. **(A)** The wild-type strain of *S.* Typhimurium carrying a plasmid expressing P*iolE::luxCDABE* or P*srfJ::luxCDABE* transcriptional fusions was grown for 24 h in LB at 37°C with aeration (non-invasive conditions). These bacteria were used to infect RAW264.7 murine macrophage-like cells and luminescence produced by intracellular bacteria was measured 2, 4, 8, and 24 h p.i. RLU, relative light units. **(B)** The wild-type strain of *S.* Typhimurium (14028) and derivatives expressing a 3xFLAG-tagged form of SrfJ in a wild-type (wt) background or in a ΔP*iolE* background were grown under non-invasive conditions and used to infect RAW264.7 cells. Expression of *srfJ* was measured 8 h p.i. by immunoblot using anti-FLAG antibodies. Anti-GroEL antibodies were used as loading control. Molecular mass markers, in kDa, are indicated on the left.

*Salmonella enterica* serovar Typhimurium is known to infect macrophages and express the T3SS2 several hours p.i. (Drecktrah et al., 2006). Since SrfJ is an effector of this secretion system, it is expected to be produced inside macrophages. To ascertain the relevance in this context of the two promoters that drive the expression of *srfJ*, *Salmonella* strains carrying P*iolE::lux* or P*srfJ::lux* transcriptional fusions were used to infect RAW264.7 macrophages. The luminescence resulting from the activity of the *lux* operon driven by P*srfJ* increased over time during the infection (**Figure 4A**). In these conditions the P*iolE* promoter was not active. Expression of *srfJ* in internalized bacteria was also studied by immunoblot using a strain of *Salmonella* that expresses a chromosomally 3xFLAG-tagged version of SrfJ. Intracellular expression was detected both in a wild-type background and in a strain lacking the distal promoter P*iolE* (**Figure 4B**). These results suggest that *srfJ* expression is induced in response to intravacuolar signals and that the induction depends specifically on the proximal promoter.

### Contribution of SrfJ to Proliferation of *Salmonella* in Macrophages

**FIGURE 5:**
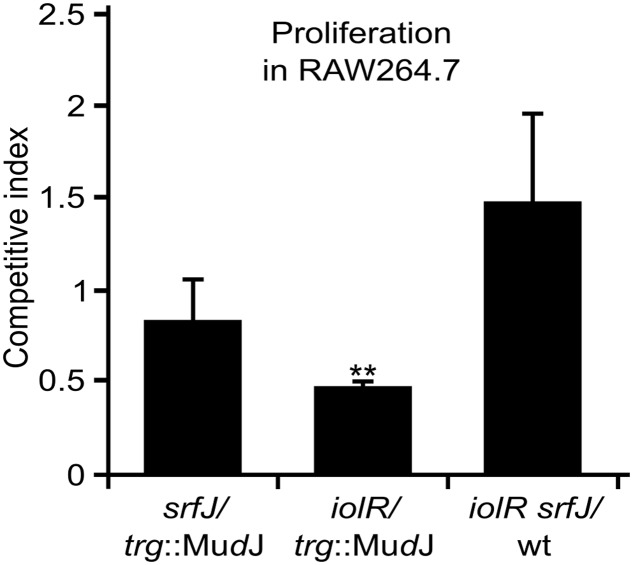
Effect of the expression of *srfJ* in intracellular proliferation. Analysis of intracellular proliferation of *srfJ*, *iolR*, and *srfJ iolR* mutants in mixed infections with a *trg*::Mu*d*J mutant or the 14028 strain (wt) used as control strains. The competitive indices are the mean from three infections. Error bars represent the standard deviations. Asterisks denote that the indices are significantly different from 1 for a *t*-test: ^∗^*P*-value < 0.05, ^∗∗^*P*-value < 0.01.

Since the *srfJ* mutant is attenuated in mice (Ruiz-Albert et al., 2002), we decided to explore the possibility that this mutant could also have a defect in survival and proliferation inside macrophages. This was assessed calculating the competitive index in RAW264.7 macrophages of the *srfJ* mutant against the *trg*::Mu*d*J strain, which is wild-type for intracellular proliferation (Segura et al., 2004). No significant defect was detected for this mutant (*P* > 0.05; **Figure 5**). We also tested the effect of a null mutation in *iolR* and we found a very significant defect in intracellular proliferation (**Figure 5**). Since the *iolR* mutation leads to derepression of *srfJ* transcription (**Figure 2**), we then measured the intracellular proliferation of the double null mutant *iolR srfJ*. Interestingly, the *srfJ* mutation suppressed the effect of the *iolR* mutation on macrophages (**Figure 5**), suggesting that the proper regulation of the expression of *srfJ* is essential for survival and/or proliferation of *S.* Typhimurium inside murine macrophages.

### Expression of *srfJ* in the Presence of Plant Extracts

**FIGURE 6:**
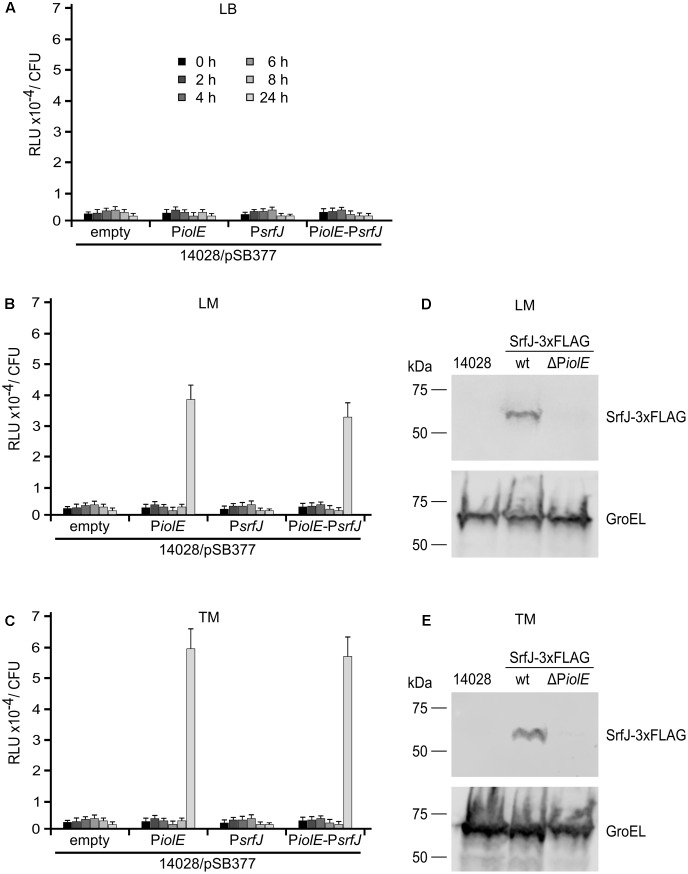
Activity of P*iolE* and P*srfJ* in media with plant extracts. Fragments of DNA containing the promoters of *iolE* (P*iolE*) or *srfJ* (P*srfJ*) or the region containing from the promoter of *iolE* to the promoter of *srfJ*, including genes *iolE* and *iolG1* (P*iolE*–P*srfJ*) were cloned into plasmid pSB377 to generate *luxCDABE* transcriptional fusions. These plasmids as well as the empty plasmid were introduced into *S.* Typhimurium strain 14028. Luminescence was measured at different time points (0, 2, 4, 6, 8, 10, and 24 h) in cultures grown in **(A)** LB, **(B)** Lettuce Medium (LM), and **(C)** Tomato Medium (TM). Bacteria were grown overnight in LB and diluted to OD_600_ 0.1 in the different test media before 0 h time point. RLU, relative light units. The wt strain of *S.* Typhimurium (14028) and derivatives expressing a 3xFLAG-tagged form of SrfJ in a wt background or in a ΔP*iolE* background were grown in LM **(D)** or TM **(E)**. Expression of *srfJ* was measured 8 h p.i. by immunoblot using anti-FLAG antibodies. Anti-GroEL antibodies were used as loading control. Molecular mass markers, in kDa, are indicated on the left.

The results obtained in macrophages confirmed our hypothesis and proved that SrfJ, as a T3SS2 effector, depends on P*srfJ*, the promoter that is induced in media imitating intravacuolar conditions. In contrast, it is more difficult to understand the physiological role of the expression of *srfJ* from the distal promoter, P*iolE*. In order to investigate the significance of the double regulation we analyzed different environments known to host *Salmonella*, one of them are plants. *Salmonella* is able to thrive and proliferate in plants, including crop plants designated for direct consumption, e.g., lettuce or tomatoes. Thus, we reasoned that since most plants produce MI, P*iolE* could be relevant in allowing transcription of *srfJ* in these alternative hosts. We therefore explored the expression of *srfJ* in response to plant signals. *Salmonella* with *lux* transcriptional fusions were grown in LB or in media supplemented with lettuce (LM) or tomato (TM) extracts. We detected high level of luminescence 24 h after the inoculation of LM or TM media with *Salmonella* carrying the long fusion P*iolE-srfJ::lux* or the P*iolE::lux* fusion. Luminescence was not detected after inoculation with a strain carrying the P*srfJ::lux* fusion (**Figures 6A–C**). Expression of *srfJ* was also studied at the protein level taking advantage of the chromosomal SrfJ-3xFLAG fusion. As shown in **Figures 6D,E**, SrfJ-3xFLAG was detected by immunoblot in extracts from bacteria grown in LM or TM for 24 h. However, the protein was not produced if P*iolE* was deleted. These results show that P*iolE* can drive expression of *srfJ* in response to plant extracts.

### Expression of *srfJ* in Plants

**FIGURE 7:**
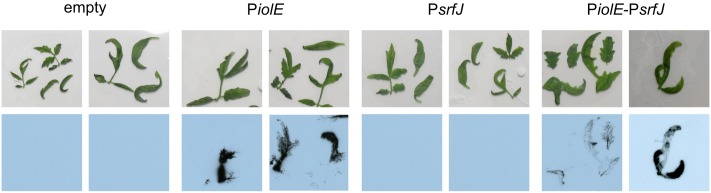
Expression in plants. *S.* Typhimurium strain 14028 carrying derivatives of plasmid pSB377 (empty, P*iolE*, P*srfJ*, P*iolE*–P*srfJ*) was used to spray 3-week-old tomato plants grown in sterile conditions. Plants were germinated in ½ MS for 1 week and grown in ¼ MS media during 2 weeks. Bacteria were grown 1 day before the infection in LM plates. Three tomato plants were spray-inoculated with bacteria suspended in 10 mM MgCl_2_ at OD_600_ 0.1. Tomato plants were imaged 2 days p.i. with an X-ray film exposed for 48 h.

The results presented above suggest that *srfJ* could be expressed during *Salmonella* colonization of plants. To evaluate this hypothesis, 3-week-old tomato plants were spray-irrigated with suspensions of wild-type *S.* Typhimurium carrying derivatives of plasmid pSB377 to generate transcriptional *luxCDABE* fusions with P*iolE*, P*srfJ*, or P*iolE–srfJ*. Tomato leaves were imaged 2 days post-inoculation using an X-ray film. Luminescence was detected in plants colonized with bacteria carrying P*iolE::lux* and P*iolE-srfJ::lux* fusions but not with P*srfJ::lux* or the empty vector (**Figure 7**). Promoters of genes encoding effectors SlrP and SteA were also tested in this system but their expression was not detected (data not shown). These results reveal that *Salmonella* expresses *srfJ* together with the MI utilization island, during colonization of a plant host.

### Contribution of SrfJ to Survival of *Salmonella* in Plants and Activation of Plant Defense Responses

**FIGURE 8:**
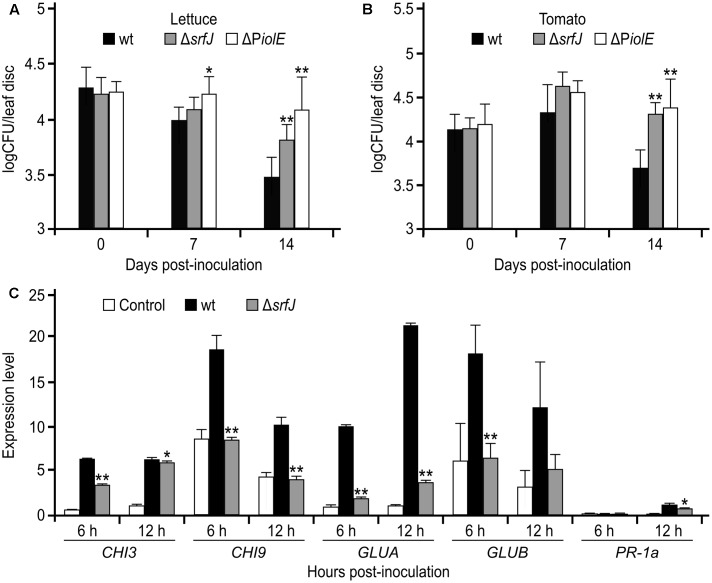
Survival of *Salmonella srfJ* mutants and activation of defense response genes in plants. *Salmonella* wt strain 14028 (wt), *srfJ* mutant, and P*iolE* mutant were syringe infiltrated onto leaves of lettuce **(A)** or tomato **(B)**. The infiltrated leaves were sampled 0, 7, and 14 days after infiltration to determine the number of CFU of *Salmonella*. The results shown are the means from six experiments. Error bars represent standard deviations. Asterisks indicate significant differences of mutants compared to wt the same day post-inoculation by Student’s *t*-test (^∗^*P* < 0.05, ^∗∗^*P* < 0.01). **(C)** *Salmonella* wt and *srfJ* mutant were used to spray 3-week-old tomato plants grown in sterile conditions. Plants were sampled 6 and 12 h post-inoculation. Relative expression levels of *CHI3*, *CHI9*, *GLUA*, *GLUB*, and *PR-1a* were assessed using quantitative RT-PCR and normalized to the expression of the house keeping gene for actin. Data are presented as mean values + standard deviations of three replicates. Asterisks indicate significant differences of mutants compared to wt by Student’s *t*-test (^∗^*P* < 0.05, ^∗∗^*P* < 0.01).

The expression of *srfJ* in plants suggested that the product of this gene could be relevant during *Salmonella* colonization of these alternative hosts. To test this hypothesis, we compared the survival of wild-type *S.* Typhimurium with the survival of the *srfJ* mutants in leaves of lettuce and tomato. Leaves were syringe infiltrated with bacterial suspensions and the CFU were counted at different time points. Interestingly, the *srfJ* null mutant showed a significantly improved survival in leaves of both plants 14 days post-inoculation (**Figures 8A,B**). Since expression of *srfJ* in plants depends specifically on the P*iolE* promoter, we also tested survival in plant leaves of a *S.* Typhimurium mutant with an intact coding sequence of *srfJ* but with a deletion of P*iolE*. As shown in **Figures 8A,B**, this mutant confirmed the results obtained with the *srfJ* mutant. These results suggest that SrfJ could be involved in the modulation of plant defense responses that could limit bacterial growth. To test this hypothesis, the expression of five genes known to be involved in tomato defense responses was studied after inoculation of tomato plants with *Salmonella* wild-type or *srfJ* mutant using qRT-PCR. Monitored genes encode an acidic extracellular chitinase (*CHI3*), a basic intracellular chitinase (*CHI9*), an acidic extracellular β-1,3-glucanase (*GLUA*), a basic intracellular β-1,3-glucanase (*GLUB*), and a PR-1 protein isoform PR-P6 (*PR-1a*) (Joosten and De Wit, 1989; Enkerli et al., 1993; Uehara et al., 2010). Interestingly, the expression of these genes was significantly lower 6 h and/or 12 h after inoculation with the *srfJ* mutant compared to the wild-type (**Figure 8C**).

## Discussion

We previously showed that SrfJ can be secreted through the T3SS2 of *S.* Typhimurium and that its expression is positively regulated by PhoP and SsrB, and negatively regulated by RcsB and IolR (Cordero-Alba et al., 2012). In this work, we show that these regulators act through two different promoters: a proximal promoter, P*srfJ*, that responds to PhoP, SsrB, and RcsB, and a distal promoter, P*iolE*, that responds to IolR (**Figure 2**).

The proximal promoter (P*srfJ*) initiate the transcription in an adenine located 33 bases upstream the *srfJ* start codon (**Figure 3C**). The three regulators that control expression from P*srfJ* are well-known relevant regulators of *Salmonella* virulence. (i) SsrB is the response regulator of a *Salmonella*-specific two-component regulatory system where the kinase SsrA detects low pH in the host vacuole through a histidine-rich periplasmic sensor domain (Mulder et al., 2015) and phosphorylates SsrB. Phosphorylated SsrB activates transcription of target genes (Deiwick et al., 1999). Positive regulation by SsrB is a common feature of SPI2 genes and other genes encoding effectors specifically secreted through T3SS2 (Xu and Hensel, 2010). (ii) The ancestral PhoQ/PhoP regulatory system is a master two-component system that regulates more than 100 genes (Zwir et al., 2005; Tran et al., 2016) in response to environmental signals including low Mg^2+^, acidic pH, and cationic antimicrobial peptides (Chamnongpol et al., 2003; Bader et al., 2005; Prost et al., 2007). Since PhoP regulates expression of *ssrA* and *ssrB* (Bijlsma and Groisman, 2005), it also regulates expression of genes in the SsrB regulon. (iii) Finally, the Rcs phosphorelay system has been shown to play an important role in virulence in mice, in particular during systemic infections (Detweiler et al., 2003; Domínguez-Bernal et al., 2004; García-Calderón et al., 2005; Erickson and Detweiler, 2006). Repression of P*srfJ* by RcsB correlates with previous reports suggesting that high level of activation of the system negatively regulates expression of SPI1 and SPI2 genes (Wang et al., 2007). Thus, this pattern of regulation indicates that P*srfJ* functions as a typical promoter of a T3SS2-related gene that responds specifically to intravacuolar signals. Consistent with this, transcription from this promoter was induced in LPM at pH 5.8, a medium that mimics intracellular conditions (**Figure 1**). More importantly, it was also induced in bacteria phagocytized by macrophages (**Figure 4**), which are conditions known to induce the expression of the T3SS2.

Regulation by IolR and MI was more puzzling. Nonetheless, we were able to show that these act through a different, distal promoter, P*iolE*. The transcription start site corresponding to this promoter is an adenine located 99 bases upstream of *iolE* (**Figure 3C**) and 2130 bases upstream of *srfJ*. Two lines of evidence support the existence of an operon encompassing *iolE*, *iolG1*, and *srfJ*: (i) RT-PCR carried out with appropriate oligonucleotide pairs on an RNA sample obtained from an *iolR*-mutant strain yielded products of the expected size (**Figure 3B**) if the three genes are transcriptionally linked. (ii) P*iolE* can drive expression of the reporter *lux* operon in response to an *iolR* mutation (**Figure 2**) or MI supplementation (not shown) from a proximal (pSB377-P*iolE*) and a distal position (pSB377-P*iolE*–P*srfJ*).

In contrast to P*srfJ*, P*iolE* does not respond to intravacuolar signals but to MI. This carbohydrate, produced by most of the plants, is important for plant growth and development (Loewus and Loewus, 1983): oxidation of MI is an important pathway in cell wall polysaccharide biogenesis (Loewus and Murthy, 2000; Loewus, 2006); inositol and derived molecules are involved in stress-related responses (Loewus and Murthy, 2000); and MI is used as a precursor of inositol-containing signaling molecules including phosphatidylinositol and phosphoinositides (Gillaspy, 2011). The presence of this carbohydrate in plant extracts explains expression of *srfJ* from P*iolE* in LM and TM (**Figure 6**). The observation that this expression is detected 24 h but not 8 h after the inoculation of the media is in agreement with a previously reported extended lag phase during the growth of *Salmonella* in the presence of MI as the sole carbon source (Kröger and Fuchs, 2009). The authors of this report exclude catabolite repression as an explanation but suggest that the *iol* genes in *Salmonella* could be under a tight repression or under the action of an additional unknown regulatory factor. Interestingly, our results suggest that expression of *srfJ* from the MI responsive promoter P*iolE* could be important during the plant colonization by *Salmonella* (**Figure 7**). This could also explain the chromosomal location of *srfJ* inside the MI island from an evolutionary point of view.

Several reports suggest the important role of *Salmonella* T3SS during plant colonization. *Salmonella* lacking T3SS1 and T3SS2, *prgH*, and *ssaV* mutants, respectively, showed reduced proliferation in syringe-infiltrated leaves of *Arabidopsis thaliana* (Schikora et al., 2011). Symptoms caused by these mutants were more pronounced in comparison with the wild-type strain, indicating that T3SSs are involved in suppressing the hypersensitive response (HR), an induced, localized cell death, which limits the spread of pathogens. Furthermore, transcriptome analyses showed that a *prgH* mutant induced stronger defense gene expression than wild-type bacteria in *Arabidopsis* seedlings (Schikora et al., 2011; Garcia et al., 2014). Similarly, experiments in *Nicotiana tabacum* have shown that the T3SS1 is essential for the active suppression of defense mechanisms by *Salmonella* (Shirron and Yaron, 2011). Interestingly, the response was different in other plants or even in different organs of the same plants: mutants lacking T3SS1 (*sipB* or *spaS*) colonized *Medicago sativa* roots, *A. thaliana* roots, and *Triticum aestivum* roots and seedlings in significantly greater numbers than the wild-type strain 14028 (Iniguez et al., 2005). Because the *sipB* mutation did not enhance colonization in a *npr1 Arabidopsis* mutant, which is defective in both salicylic acid (SA)-dependent and SA-independent defense responses (Ton et al., 2002), the authors concluded that T3SS1 is involved in the induction of both kinds of plant responses. In contrast, another study concluded that T3SS1 and T3SS2 were not involved in suppression of plant defenses in *Nicotiana benthamiana* leaves (Meng et al., 2013). These discrepant results indicate that the exploration of a variety of experimental conditions and host models will be necessary to ascertain the role of *Salmonella* T3SSs and particular effectors in plants. Interestingly, although *Salmonella*-mediated delivery of effector proteins into plant cells have not been shown yet (García and Hirt, 2014), effectors SseF and SspH2 were able to trigger cell death through resistance-gene-mediated signaling in *N. benthamiana* when heterologously delivered using *Agrobacterium tumefaciens* or *Xanthomonas campestris* (Üstün et al., 2012; Bhavsar et al., 2013).

An important aspect of this work was the analysis of the contribution of SrfJ to the survival of *Salmonella* inside animal and plant hosts. Our results suggest that the expression of this effector at the appropriate time is a relevant factor in the interaction of *Salmonella* with mice macrophages and with lettuce and tomato leaves: (i) The results obtained with the *iolR* mutant and the *iolR srfJ* double mutant indicate that the ectopic production of SrfJ caused by the absence of the IolR repressor decreases survival/proliferation of *Salmonella* inside RAW264.7 macrophages (**Figure 5**). (ii) The lack of production of SrfJ in plants caused by a deletion of the coding sequence of *srfJ* or a deletion of the distal promoter P*iolE* leads to an improved survival of *Salmonella* in plants (**Figure 8**). This result is in line with the reduced defense gene activation displayed by the *srfJ* mutant compared to the wild-type (**Figure 8C**) and suggests that SrfJ could act in this system as an avirulence protein (Mansfield, 2009). These proteins are effectors of plant pathogens that, in the course of plant-pathogen co-evolution, have been recognized by plant receptors to activate defense responses. As such, SrfJ could have a virulent role in other sensitive plant strains or species.

Additional experiments will be needed to ascertain if SrfJ expressed in *Salmonella* during plant colonization can be secreted through T3SS2. In this context, it should be noted that in our experimental model P*srfJ* was not active during plant colonization with *Salmonella*. This result suggests that, under these conditions, the SsrB regulon, including, SPI2, would not be expressed and therefore SrfJ, although expressed from P*iolE*, would not be secreted through T3SS2. Nevertheless, the phenotype of the *ssaV* mutant noted above (Schikora et al., 2011) argues for the expression of this system at some point of the colonization of plants. Our previous results showed that translocation into macrophages was specifically T3SS2-dependent (Cordero-Alba et al., 2012), and here we have shown that P*srfJ* is the only promoter that drives expression of *srfJ* inside these cells (**Figure 4**). However, secretion of SrfJ through T3SS1 is another interesting possibility that could be explored in different cell types or hosts. Several effectors can be secreted through both systems. For example, for PipB2, considered as a T3SS2 effector, we have previously shown the possibility of translocation through T3SS1 into a variety of cell types (Baisón-Olmo et al., 2012). Specificity of secretion is achieved, at least for some effectors, simply by coexpression between the particular T3SS and its effectors. An example is SseK1: when expressed from a constitutive promoter it can be secreted through T3SS1 at earlier time points p.i. than when expressed from its own promoter (Baisón-Olmo et al., 2015). Coexpression of T3SS1 and *srfJ* from P*iolE* may take place in plants due to the presence of MI, making it possible the delivery of the effector through this way. A recent report suggests that *S.* Typhimurium is unable to translocate T3SS effectors into cells of beet roots or pepper leaves (Chalupowicz et al., 2017). However, there is the possibility of translocation in other plant systems. Alternatively, some effectors could be secreted and exert their action in the apoplast rather than inside plant cells.

The function of SrfJ inside the host is presently unknown. Its amino acid sequence and its structure (Kim et al., 2009) suggest that it may have glucosylceramidase activity. This enzymatic activity catalyzes hydrolysis of glucosylceramide into glucose and ceramide, the simplest member of the family of sphingolipids. These lipids not only represent essential structural elements of membranes, but several members of this family, including ceramide, are also secondary messenger molecules that regulate intra- and intercellular processes (Ilan, 2016). Ceramides can affect cellular proliferation, differentiation, cell death, insulin resistance, oxidative stress, and inflammation (Pandey et al., 2007; Saddoughi and Ogretmen, 2013; Kogot-Levin and Saada, 2014; Kuzmenko and Klimentyeva, 2016). Glycosphingolipids are membrane components that can affect numerous cellular events including homeostasis, adhesion growth, motility, apoptosis, proliferation, stress, and inflammatory responses (Ilan, 2016). Interestingly, glucosylceramide is the only glycosphingolipid that plants, fungi, and animals have in common (Warnecke and Heinz, 2003). Glucosylceramide is important in animals for the activation of antigen-presenting cells, induction of Th1 and Th7 responses, and neutrophil recruitment (Pandey et al., 2012). There is less information about the functions of glucosylceramide in plants, but it has been suggested to be necessary for normal Golgi-mediated protein trafficking (Melser et al., 2010, 2011). A more recent report has shown that null mutants for glucosylceramide synthase failed to develop beyond the seedling stage and that glucosylceramide is critical for cell-type differentiation and organogenesis (Msanne et al., 2015). Future research should focus on the relevance and consequences of the putative effect of SrfJ on this important lipid target both in animal and plant cells.

## Author Contributions

All authors listed above have made a substantial, direct and intellectual contribution to the work, and approved it for publication.

## Conflict of Interest Statement

The authors declare that the research was conducted in the absence of any commercial or financial relationships that could be construed as a potential conflict of interest.
